# Medium-term impacts of poultry litter application on potentially toxic elements in soil under subtropical conditions in southern Brazil

**DOI:** 10.1007/s11356-026-37995-3

**Published:** 2026-06-30

**Authors:** Débora de Mello, Mário M. Tagliari, Miguel A. Sierra-Garcia, Paula Bermeo-Fúquene, Jorge L. Zanatta, Luis C. Cassol, Larissa M. S. Tonial, Thiago O. Vargas

**Affiliations:** 1 Post-Graduate Program in Agronomy, Federal Technological University of Parana–Pato Branco Campus, Programa de Pós-Graduação Em Agronomia, Universidade Tecnológica Federal do Paraná–Campus Pato Branco, Via Do Conhecimento, Km 01, Pato Branco City, Paraná State 85503-390 Brazil; 2 Nataima Research Center, Colombian Corporation for Agricultural Research (AGROSAVIA), Km 09 Espinal–Ibagué Road, Tolima City, 733529 Colombia

**Keywords:** Poultry litter, Potentially toxic elements, Soil pollution, Long-term field experiment, Organic fertilizer, Zinc accumulation, Subtropical soils

## Abstract

Poultry litter is widely applied as an organic fertilizer, yet concerns remain regarding the cumulative input and potential accumulation of potentially toxic elements (PTEs) in agricultural soils. This study evaluated the medium-term effects of repeated poultry litter application on pseudo-total concentrations and cumulative inputs of arsenic (As), cadmium (Cs), chromium (Cr), copper (Cu), nickel (Ni), and zinc (Zn) in an acidic dystrophic Red Latosol under subtropical conditions in southern Brazil. An eight-year field experiment (2011–2018) evaluated four annual poultry litter rates (0, 4, 8, and 12 t ha⁻^1^) combined with dolomitic limestone application under a randomized complete block split-plot design. Baseline soil characterization, cumulative PTE mass balance, and linear mixed-effects models were integrated to assess treatment responses and environmental relevance. Despite continuous inputs, no significant accumulation of As, Cd, Cr, Cu, or Ni was detected within the evaluated 0–20 cm soil layer, likely reflecting low cumulative additions relative to initial soil stocks and soil retention processes. In contrast, Zn showed significant dose-dependent accumulation in the surface layer (0–10 cm), consistent with its higher cumulative input from poultry litter. Dolomitic limestone application had no detectable effect on PTE dynamics during the evaluated period, probably due to the limited increase in soil pH. Although all PTE concentrations remained below Brazilian regulatory thresholds, cumulative Zn inputs represented the largest proportional increase relative to the initial soil pool, highlighting Zn as a sensitive indicator for monitoring repeated organic amendment applications. These findings emphasize the importance of combining baseline characterization, mass balance approaches, and continued monitoring to improve environmental risk assessments of organic waste reuse in subtropical agroecosystems.

**Supplementary Information:**

The online version contains supplementary material available at 10.1007/s11356-026-37995-3.

## Introduction

The global expansion of poultry production has led to the generation of large volumes of poultry litter, a heterogeneous residue composed of animal excreta, bedding material, feed residues, and feathers. In countries with intensive poultry production systems, such as Brazil, poultry litter has been widely reused as an organic soil amendment due to its agronomic benefits, including improvements in soil organic matter, structure, water retention, and nutrient supply (Lin et al. 2018; Watts et al. 2010; Xiao et al. 2022). This practice is embedded within broader circular economy strategies aimed at recycling nutrients and reducing dependence on synthetic fertilizers.

However, the agricultural reuse of poultry litter also represents a long-term environmental management challenge. Poultry litter contains potentially toxic elements (PTEs), such as arsenic (As), cadmium (Cd), chromium (Cr), copper (Cu), nickel (Ni), and zinc (Zn), largely originating from feed additives, veterinary supplements, and mineral impurities (De Souza et al. 2019; Okada et al. 2024).

The environmental behavior of PTEs in amended soils is highly site-specific and governed by interactions among soil properties, climate, management practices, and cumulative input rates. Previous studies have reported contrasting outcomes, ranging from significant surface accumulation—particularly for Zn and Cu—to limited or undetectable changes over time (Mitchell and Tu 2006; Ashjaei et al. 2011; Xiao et al. 2022). Such discrepancies are often linked to differences in soil pH, texture, organic matter content, and rainfall regimes, factors that strongly influence metal retention, mobility, and detectability (Appel and Ma 2002; Foust et al. 2018).

One critical limitation of the existing literature is that many field studies lack baseline soil PTE characterization before treatment establishment or rely on relatively short experimental durations. Without baseline reference values, it becomes difficult to distinguish treatment-driven changes from natural spatial variability or background geochemical trends. Furthermore, claims of leaching or immobilization are often inferred rather than directly demonstrated, particularly in tropical soils where high rainfall, advanced weathering, and acidic conditions may promote both vertical redistribution and biological removal of PTEs.

In acidic soils, cationic PTEs generally exhibit higher solubility, which may reduce surface accumulation without necessarily producing detectable increases at shallow subsurface depths within medium-term monitoring windows. Consequently, the absence of measurable accumulation should not be interpreted as evidence of environmental safety, but rather as an indication of system buffering and limited analytical detectability under realistic agronomic conditions.

Against this background, there remains a clear need for medium- to long-term field studies explicitly framed within a systems perspective, integrating baseline soil characterization, cumulative mass inputs, depth-resolved monitoring, and cautious interpretation of nonsignificant results. Therefore, this study presents an eight-year field experiment conducted in an acidic dystrophic Red Latosol in southern Brazil, designed to evaluate the system-level response of soil PTE concentrations to continuous poultry litter application under realistic management conditions. Specifically, we tested the hypotheses that (i) Zn accumulates in surface soil layers due to its relatively high cumulative input and intermediate retention capacity and (ii) cumulative inputs of As, Cd, Cr, Cu, and Ni over eight years are insufficient to produce detectable increases in pseudo-total concentrations in the 0–20 cm soil layer under acidic tropical conditions. By emphasizing baseline conditions, temporal resolution, and system constraints, this study aims to contribute to more nuanced environmental risk assessments of organic amendments in tropical agroecosystems.

## Materials and methods

### Study area and soil characterization

**Fig. 1:**
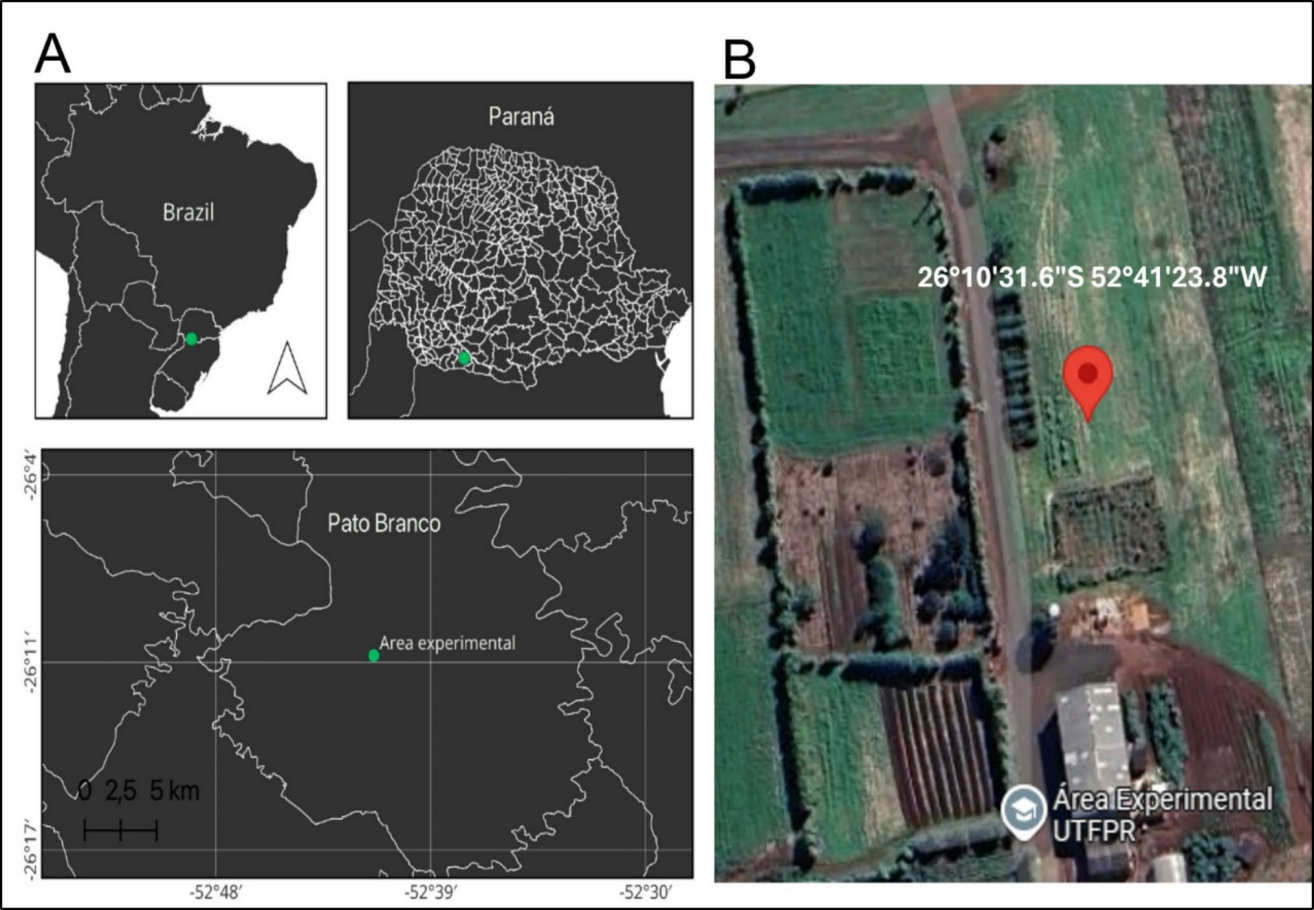
**A** Location of the experimental area in Southwestern Paraná (Pato Branco), Brazil. **B** Location of the experiment (“*Área experimental*”) at *Universidade Tecnológica Federal do Paraná* (UTFPR), Campus Pato Branco, Brazil

The experiment was conducted from 2011 to 2018 at the experimental station (i.e., *Área Experimental*) of *Universidade Tecnológica Federal do Paraná* (UTFPR), Pato Branco, Brazil (26° 10′ 33″ S, 52° 41′ 23″ W; Fig. 1). The region has a humid subtropical climate (Köppen Cfa), with an annual mean temperature of 18.5 °C and precipitation of 1.931 mm. Prior to treatment establishment (2011), baseline soil samples were collected from the 0–20 cm layer to determine initial PTE concentrations and soil physical properties, ensuring reliable temporal comparisons. Initial soil properties were organic matter 66.3 g dm⁻^3^, available P 20.2 mg dm⁻^3^, K 0.49 cmolc dm⁻^3^, Ca 4.70 cmolc dm⁻^3^, Mg 2.63 cmolc dm⁻^3^, Al 0.25 cmolc dm⁻^3^, pH (CaCl₂) 4.73, CEC 15.1 cmolc dm⁻^3^, and base saturation 51.8%. Soil texture consisted of 540 g kg⁻^1^ sand, 180 g kg⁻^1^ silt, and 280 g kg⁻^1^ clay, characterizing a sandy clay loam. Baseline pseudo-total concentrations (mg kg⁻^1^) were: As 9.8, Cd 0.7, Cr 41.5, Cu 58.9, Ni 12.4, and Zn 43.2.

### Experimental design and treatments

A randomized complete block split-plot design with four replications was established. The main plots received four doses of dolomitic limestone (0, 2, 4, and 8 t ha⁻^1^ with 82% neutralizing value) applied once in 2015 to address soil acidity. Each main plot was subdivided into four subplots, which were treated annually from 2011 to 2018 with poultry litter at four rates (0, 4, 8, and 12 t ha⁻^1^, applied on a wet weight basis), resulting in a full factorial combination of treatments. Poultry litter was surface-applied and subsequently incorporated into the top 10 cm of soil using a disc harrow. Poultry litter was chemically characterized annually (2011–2018). Mean concentrations (± SD, dry basis) were reported on a dry weight basis (Table 1).

**Table 1:** Average composition of N, P, K, As, Cd, Cr, Cu, Ni, and Zn of collected poultry litter from 2011 to 2018 (dry weight basis)

Macronutrients						
Elements		N	P	K		
Unit		g kg^−1^				
Values		25.9	15.9	39.5		
PTEs						
Elements	As	Cd	Cr	Cu	Ni	Zn
Unit	mg kg^−1^					
Values	2.80	0.38	15.72	278.25	12.62	342.17

Cumulative PTE inputs derived from poultry litter application were calculated to estimate the total mass of each element added to the soil during the experimental period. For each poultry litter dose, cumulative elemental inputs were calculated according to the equation below (1).

1 $$\text{Cumulative PTE input} \left({kg}^{-1}\right)=\text{PL rate}\ast \mathrm{PTEPL} \ast \mathrm{Y} \ast \mathrm{CF}$$

where *PL rate* represents the annual poultry litter application rate (t ha⁻^1^ yr⁻^1^), *PTEPL* is the mean concentration of each element in poultry litter (mg kg⁻^1^, dry weight basis), *Y* represents the number of consecutive application years (eight years), and CF is the unit conversion factor required to convert mg to kg. To evaluate the environmental relevance of these cumulative poultry litter-derived inputs, initial soil PTE stocks in the 0–20 cm layer were estimated considering soil bulk density and sampling depth according to Eq. (2):

2 $$\text{Soil PTE stock} \left(kg\;{ha}^{-1}\right)=\text{PTE soil} \ast \mathrm{BD} \ast \mathrm{D} \ast 10$$

where *PTEsoil* represents the pseudo-total concentration of each element measured before treatment establishment (mg kg⁻^1^), *BD* is soil bulk density (Mg m⁻^3^), *D* is soil depth (cm), and *10* is the conversion factor. Because soil bulk density was not determined during the initial characterization, soil PTE stocks were estimated using a representative bulk density value of 1.25 Mg m⁻^3^, based on soil texture, organic matter content, and typical values reported for cultivated Brazilian Oxisols under similar management conditions. These estimates were used exclusively to provide a mass balance interpretation of cumulative PTE inputs relative to the initial soil elemental pool.

The relative contribution of poultry litter-derived PTE inputs to the initial soil pool was then calculated as Eq. (3):

3 $$\text{Relative contribution} \left(\%\right)=\left(\frac{\text{cumulative PTE input}}{\text{initial soil PTE stock}}\right)\ast 100$$

The cropping system initially followed wheat (winter) and soybean (summer) rotations from 2011 to 2015, transitioning to black oat (IAPAR 61 variety at 60 kg seeds ha⁻^1^) and maize (Pioneer 30F53 VYH hybrid at 85,000 plants ha⁻^1^) from 2016 to 2018. To ensure balanced nutrition across all plots, mineral fertilizers were applied to all treatments (including the control) based on soil test recommendations to supplement nutrients not sufficiently supplied by the poultry litter, aiming to achieve equivalent crop nutrient availability and isolate the effect of PTE inputs from the litter. Cumulative PTE inputs were expressed as total elemental loads added per unit area (kg ha⁻^1^) rather than as soil concentrations, allowing direct comparison between poultry litter-derived inputs and the existing soil PTE pool. Over the eight-year application period, cumulative PTE inputs increased proportionally with poultry litter rates (Table 2). At the highest application rate (12 t ha⁻^1^ yr⁻^1^), total additions reached 0.27 kg ha⁻^1^ As, 0.04 kg ha⁻^1^ Cd, 1.51 kg ha⁻^1^ Cr, 26.71 kg ha⁻^1^ Cu, 1.21 kg ha⁻^1^ Ni, and 32.85 kg ha⁻^1^ Zn. When these inputs were compared with the initial soil PTE stocks in the 0–20 cm layer, Zn represented the greatest relative increase, followed by Cu, whereas As, Cd, Cr, and Ni accounted for only small additions relative to the pre-existing soil pools. This mass balance approach provides a broader assessment of the potential long-term contribution of poultry litter to soil PTE accumulation.

**Table 2:** Cumulative potentially toxic element (PTE) inputs from poultry litter application during the eight-year experimental period (2011–2018), estimated initial soil PTE stocks (0–20 cm), and relative contribution of the highest poultry litter rate (12 t ha⁻^1^ yr⁻^1^) to the initial soil elemental pool

Potentially toxic elements (PTE)	4 t ha⁻^1^	8 t ha⁻^1^	12 t ha⁻^1^	Initial soil stock (0–20 cm)	Contribution at highest dose (12 t ha⁻^1^)
	kg ha⁻^1^	kg ha⁻^1^	kg ha⁻^1^	kg ha⁻^1^	%
As	0.09	0.179	0.269	24.5	1.1
Cd	0.012	0.024	0.036	1.75	2.2
Cr	0.503	1.006	1.509	103.8	1.5
Cu	8.91	17.81	26.71	147.3	18.1
Ni	0.404	0.808	1.212	31	3.9
Zn	10.95	21.9	32.85	108	30.4

*Because soil bulk density was not determined during the initial characterization, soil PTE stocks were estimated using a representative bulk density value of 1.25 Mg m⁻^3^, based on the soil texture, organic matter content, and typical values reported for cultivated Brazilian Oxisols under similar management conditions. These estimates were used exclusively to provide a mass balance interpretation of cumulative PTE inputs relative to the initial soil elemental pool

### Soil sampling

Soil samples were collected in October 2017 and October 2018, yielding a total of 256 soil samples, at depths of 0–10 and 10–20 cm. Pseudo-total PTE concentrations were determined using USEPA method 3051 A, followed by ICP-OES analysis. Quality control included certified reference materials and procedural blanks, with recoveries between 85 and 110%. The absolute control treatment (0 t ha⁻^1^ poultry litter and 0 t ha⁻^1^ dolomitic limestone) was used as a reference condition to characterize background PTE concentrations in the experimental area. Reference values for the control treatment across sampling years and soil depths are provided in Supplementary Table S1. These values were used only for contextual interpretation of temporal patterns, whereas treatment effects and statistical inference were evaluated using the complete experimental dataset described below.

Samples were air-dried, ground using a mortar and pestle, and passed through a 2.0-mm sieve for general analysis and a 0.5-mm sieve for PTE analysis at the Soil Chemistry and Fertility Laboratory at UTFPR, Pato Branco–Paraná, Brazil. The prepared samples were stored for subsequent digestion and quantification of chemical parameters and potentially toxic elements (PTEs).

### Quantification of PTEs

The pseudo-total extraction of potentially toxic elements (PTEs) was performed using the US EPA 3051 A method to determine elements potentially available under strong acidic conditions. For each soil sample (0.5 g), 6.0 mL of concentrated HNO_₃_ (P.A. grade) was added to the digestion tubes. The samples were heated to 145 °C for 45 min in a digestion block equipped with a cold finger reflux system to prevent volatile losses. Following cooling, 2.5 mL of H_2_O_2_ was added to complete oxidation, with subsequent reheating at 145 °C for 15 min, resulting in a total digestion time of 60 min. The digested solutions were filtered through quantitative filter paper (blue ribbon), transferred to Falcon flasks, diluted to 7 mL with ultrapure water (18.2 MΩ·cm resistivity), and stored at 4 °C until analysis.

Elemental quantification (i.e., pseudo-total levels of As, Ni, Cd, Cr, Cu, and Zn) was conducted using inductively coupled plasma optical emission spectrometry (ICP-OES; Varian Agilent 720-ES) at the Soil Mineralogy Laboratory of *Universidade Federal do Paraná* (UFPR). Instrument calibration was performed at element-specific wavelengths: As (188.9 nm), Cd (214.4 nm), Cr (267.7 nm), Cu (327.3 nm), Ni (231.6 nm), and Zn (213.8 nm). A multielement calibration curve (0.1–1000 mg L⁻^1^) was prepared in matrix-matched solutions, with method detection limits of 0.01 mg L⁻^1^ for all analytes. Quality control included analyzing certified reference materials and reagent blanks with each batch of samples. Recovery rates for all PTEs ranged between 85 and 110%. Final PTE concentrations were calculated using the equation below (4).

4 $$FV\left({{\mathrm{m}\mathrm{g}\;\mathrm{k}\mathrm{g}}^{-1})=L \left[({\mathrm{m}\mathrm{g}\;\mathrm{L}}^{-1}\right)-B ({\mathrm{m}\mathrm{g}\;\mathrm{L} }^{-1})]\times\;FD\;(\mathrm{m}\mathrm{L}\;\ \mathrm{g}}^{-1}\right)$$

*FV* is the final PTE concentration in the soil sample, *L is the sample digest value, *FD* is the dilution factor expressed as the ratio between the final digest value and the sample mass, and *B* is the concentration measured in the reagent blank value.

### Statistical analysis

Statistical analyses were conducted in R (version 4.3.3; R Core Team 2025) through the RStudio integrated development environment (Posit Team 2026). Linear mixed-effects models were used to evaluate the effects of poultry litter application, dolomitic limestone amendment, and soil depth on pseudo-total PTE concentrations. For each sampling year (2017 and 2018), separate models were fitted for each PTE (As, Cd, Cr, Cu, Ni, and Zn). poultry litter rate, dolomitic limestone dose, soil depth (0–10 and 10–20 cm), and their interactions were included as fixed effects. To account for the randomized complete block split-plot experimental design, experimental blocks and limestone main plots nested within blocks were included as random effects. The general model structure was: PTE concentration ~ poultry litter × limestone × soil depth + (1|block/limestone plot), where poultry litter treatments corresponded to split-plots and dolomitic limestone treatments corresponded to main plots. Model significance was assessed using likelihood ratio tests (LRT; *α* = 0.05) by comparing nested models. Model assumptions were evaluated through residual diagnostics, including inspection of residuals versus fitted values to assess homogeneity of variance and quantile–quantile (*Q*–*Q*) plots to verify residual normality. When significant treatment effects were detected (*p* < 0.05), post hoc comparisons among treatments were performed using estimated marginal means followed by Tukey adjustment for multiple comparisons. Baseline soil PTE concentrations were used exclusively as reference values to characterize initial soil conditions and were not included as model covariates to avoid overparameterization.

Data were analyzed using the *easyanova* package (Arnhold 2013) for analysis of variance procedures, *lme4* (Bates et al. 2015) for fitting linear mixed-effects models, *emmeans* (Lenth and Piaskowski 2026) for estimated marginal means and post hoc multiple comparisons, and *ggplot2* (Wickham 2016) for graphical visualization.

## Results

Baseline soil characterization before treatment establishment indicated that initial PTE concentrations were within the expected range for the experimental area, providing a reference condition for evaluating temporal changes after repeated poultry litter applications. Similarly, PTE concentrations observed in the absolute control treatment during the 2017 and 2018 evaluations showed values comparable to baseline conditions, supporting the interpretation of treatment responses against natural background variability (Supplementary Table S1).

**Fig. 2:**
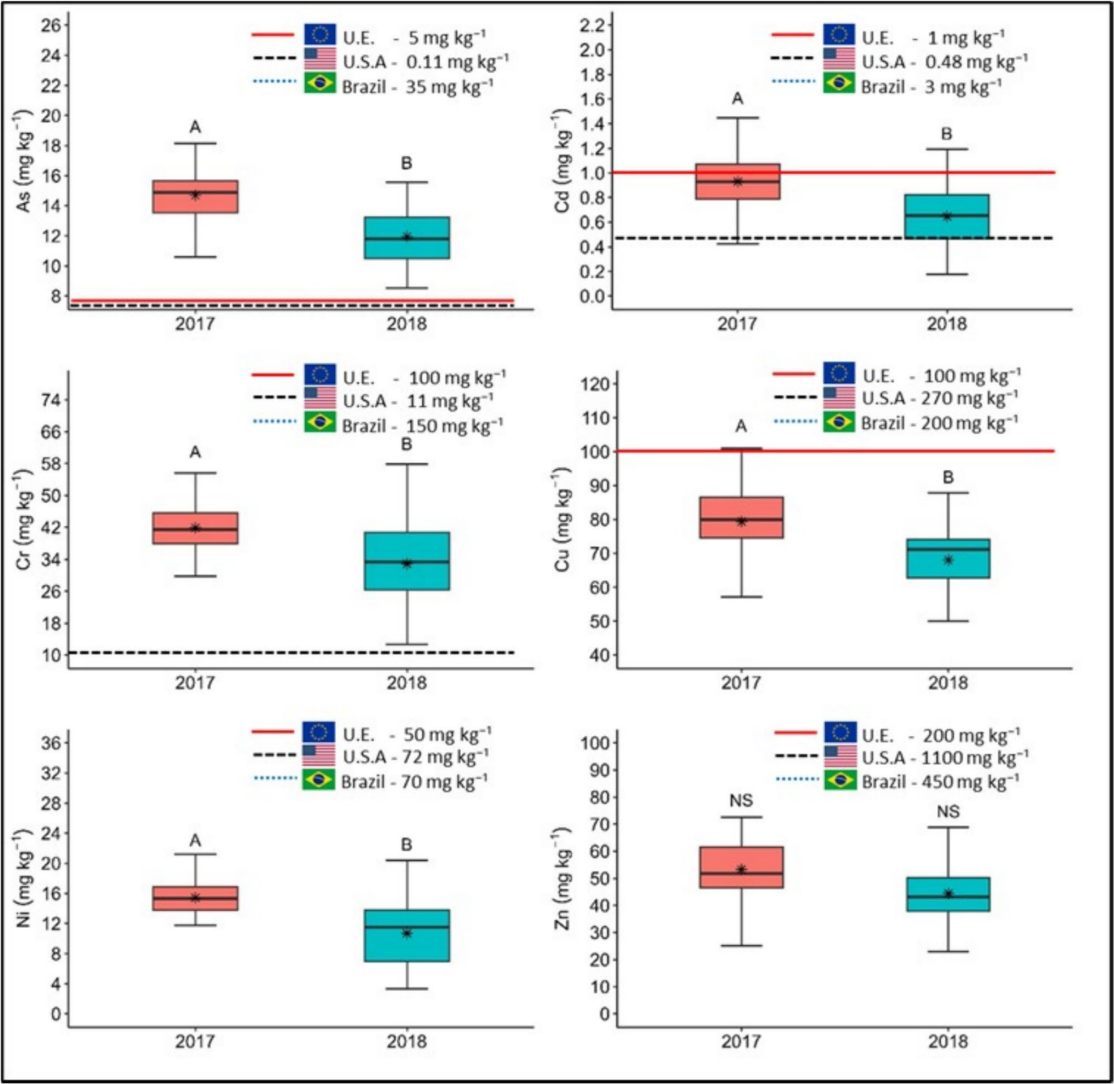
Differences in the concentrations (mg kg^−1^) of potentially toxic elements (PTEs): arsenic (As), cadmium (Cd), chromium (Cr), copper (Cu), nickel (Ni), and zinc (Zn), between the years 2017 (orange box) and 2018 (blue box) and maximum values allowable in soil for agriculture according to Brazil, USA, and European Union legislations (purple, green and red dashed lines, respectively). Different letters represent statistical significance according to the Tukey test (two-way ANOVA, *p* < 0.05), NS: not significant. An asterisk represents the mean values. European Union values: As (Fendrich et al. 2024), Cd (Ballabio et al. 2024), Cu (Ballabio et al. 2018), Cr, Ni (Tóth et al. 2016), and Zn (Van Eynde et al. 2023). Brazilian values: (CONAMA 2009). The absence of countries’ lines indicates that values registered in both years are above or below the metrics pre-established by each country

Comparisons between sampling years revealed significant differences in PTE concentrations in the 0–20 cm soil layer. Across treatments, pseudo-total concentrations of As, Cd, Cr, Cu, and Ni were significantly lower in 2018 than in 2017 (*p* < 0.05), whereas Zn showed no significant interannual change (*p* > 0.05; Fig. 2), although a numerical decrease of approximately 15–20% was observed across treatments and depths. This pattern indicates high temporal variability for Zn but no consistent net reduction between years. Also, all values remained below Brazilian regulatory thresholds (Fig. 2). In contrast, Zn did not show a significant decrease between the studied years (*p* > 0.05, Fig. 2). Compared to national legislations in 2017, its concentrations under 8–12 t ha⁻^1^ poultry litter only exceeded European guidelines, but it remained below US EPA limits (Fig. 2). Neither dolomitic limestone doses nor their interaction with poultry litter significantly affected any element (Table 3).

**Table 3:** Two-way ANOVA showing *F* values of potential toxic elements (PTEs): As, Cd, Cr, Cu, Ni, and Zn in dolomitic limestone, poultry litter, and the interaction (Dolomitic limestone X poultry litter) between years 2017 and 2018. Soil was sampled between 10 and 20 cm in depth

	PTE (mg kg⁻^1^)					
Year	2017					
	As	Cd	Cr	Cu	Ni	Zn
Dolomitic limestone	1.97ⁿˢ	1.97ⁿˢ	0.61ⁿˢ	0.64ⁿˢ	0.99ⁿˢ	0.29ⁿˢ
Poultry litter	0.10ⁿˢ	0.95ⁿˢ	0.51ⁿˢ	0.94ⁿˢ	0.53ⁿˢ	7.39***
Interaction	0.77ⁿˢ	0.78ⁿˢ	0.27ⁿˢ	0.63ⁿˢ	0.84ⁿˢ	0.68ⁿˢ
Year	2018					
	As	Cd	Cr	Cu	Ni	Zn
Dolomitic limestone	2.19ⁿˢ	0.15ⁿˢ	0.18ⁿˢ	1.90ⁿˢ	3.32ⁿˢ	1.34ⁿˢ
Poultry litter	0.22ⁿˢ	0.62ⁿˢ	0.38ⁿˢ	1.99ⁿˢ	2.05ⁿˢ	2.69ⁿˢ
Interaction	1.29ⁿˢ	1.30ⁿˢ	1.09ⁿˢ	0.64ⁿˢ	0.36ⁿˢ	0.08ⁿˢ

^ns^Nonsignificant

***p(*F*) < 0.001

Poultry litter rate significantly affected Zn concentrations in the 0–10 cm layer, with mean values of 59.0 and 64.4 mg kg⁻^1^ at 8 and 12 t ha⁻^1^ (Fig. 2; Table S2), respectively, compared to 42.1 mg kg⁻^1^ in the control (Tukey test, *p* < 0.05). No significant effects were observed for As, Cd, Cr, Cu, or Ni, and dolomitic limestone had no detectable influence on any PTE. All 2018 values became statistically indistinguishable (*p* > 0.05) despite some concentrations approaching EU limits for As and Cd. Mean values (indicated by asterisks in Fig. 2) showed consistent spatial patterns across regulatory thresholds.

**Fig. 3:**
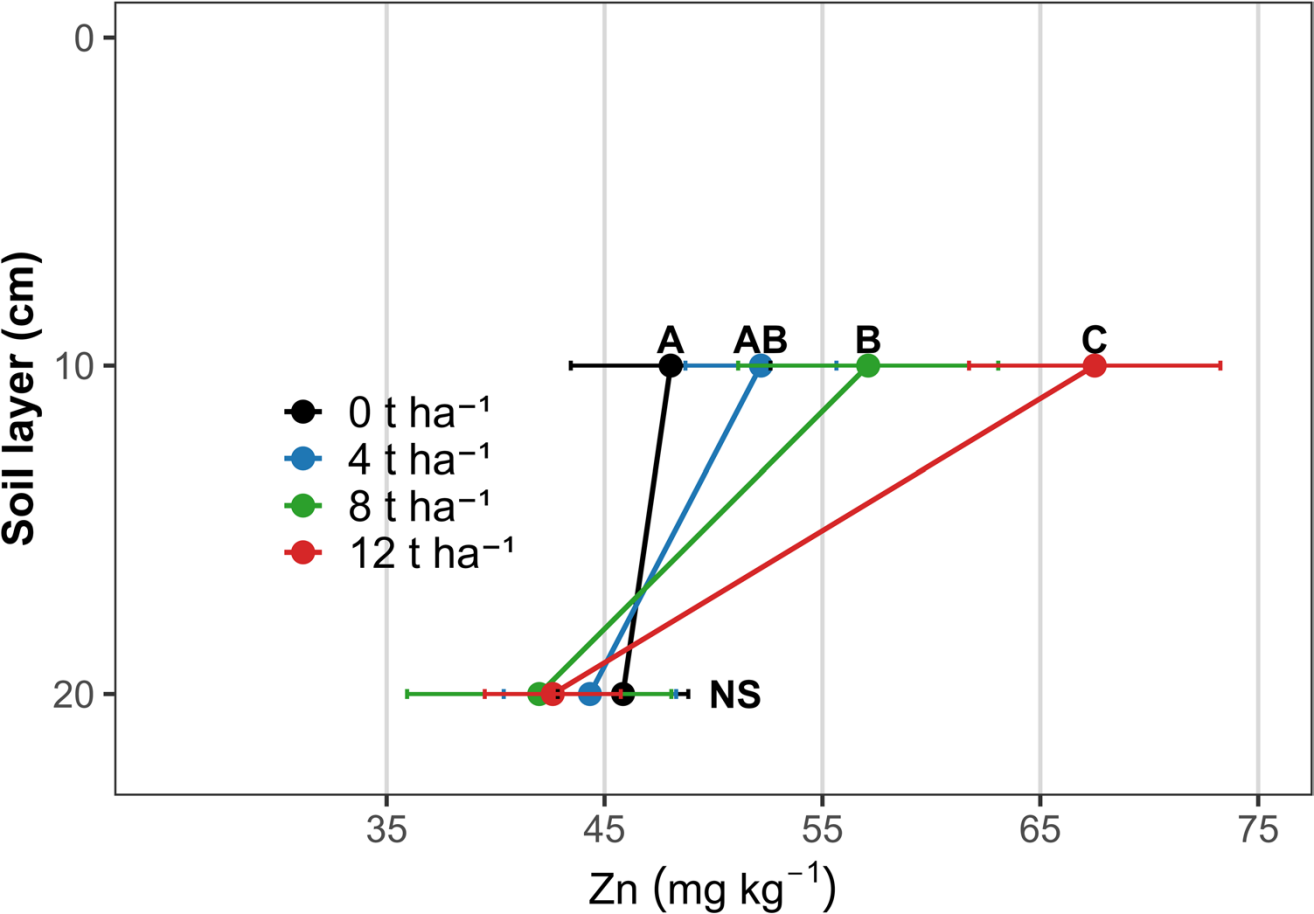
Distribution of Zn concentrations in the soil layer as affected by poultry litter doses: 0 t ha^−1^—black; 4 t ha^−1^—blue; 8 t ha^−1^—green; and 12 t ha^−1^—red. Error bars represent the standard error of the mean calculated from four replicates evaluated across the 2017 and 2018 sampling years. Different letters indicate significant differences among poultry litter rates within the 0–10 cm soil layer according to estimated marginal means followed by Tukey adjustment (*p* < 0.05). No significant differences were detected among treatments in the 10–20 cm layer (NS), indicating that Zn accumulation was mainly restricted to the surface soil layer

Between 2017 and 2018, the Zn concentration did not change in all treatments. In addition, for the mean values recorded in 2017 and 2018, significant increases in the first soil’s 10-cm depth when 12 t ha^−1^ were added (64.37 mg kg^−1^), followed by 59.05 mg kg^−1^ with the addition of 8 t ha^−1^. Poultry litter applications thus significantly affected zinc distribution (*p* < 0.05), indicating clear dose–response relationships to specific surface soils compared to control (42.11 mg kg^−1^). We have found no differences between 4 and 0 t ha^−1^, with 52.14 and 42.11 mg kg^−1^, respectively (Fig. 3).

Dolomitic limestone amendments (0–8 t ha⁻^1^) showed no significant influence on PTE concentrations (Table 3), with *F*-values ≤ 2.19 and *p* > 0.05 for all elements. Similarly, limestone × litter interactions were nonsignificant (*p* > 0.05). Vertical distribution patterns were evident for zinc, with surface layers (0–10 cm) containing 30–40% higher concentrations than subsurface layers (10–20 cm) under high litter applications (Table S2). The complete dataset of pseudo-total PTE concentrations according to year, amendment source, application rate, and soil depth is provided in Supplementary Table S2. To improve interpretation, the main results focus on the treatment effects identified by the mixed-effects models and post-hoc comparisons. Overall, repeated poultry litter application did not result in significant accumulation of As, Cd, Cr, Cu, or Ni in the evaluated soil layers. In contrast, Zn showed a significant response to poultry litter application, with higher concentrations observed under the highest application rates, particularly in the surface soil layer (0–10 cm) (Fig. 3). Dolomitic limestone application did not consistently affect soil PTE concentrations during the evaluated period.

## Discussion

This study demonstrates that under realistic poultry litter application rates, medium-term changes in pseudo-total concentrations of most PTEs are difficult to detect in acidic tropical soils. The absence of a statistically significant interannual change in Zn, despite numerical fluctuations, reinforces its role as a relatively stable surface-accumulating element under repeated poultry litter application. Also, the absence of accumulation for As, Cd, Cr, Cu, and Ni should not be interpreted as evidence of confirmed leaching, but rather as a reflection of low cumulative inputs relative to background concentrations and soil buffering capacity. For example, the total Cd input over eight years (36.5 g ha⁻^1^) is small relative to natural soil Cd pools, making measurable increases in the 0–20 cm layer unlikely. Similar reasoning applies to Cu and Ni, despite their higher absolute inputs.

The sandy clay loam texture of the soil, combined with high rainfall conditions, may have favored greater mobility of more soluble PTE fractions; however, vertical transport below the evaluated 20 cm soil layer was not directly assessed in this study. Therefore, the observed interannual reductions should be interpreted as changes in pseudo-total concentrations within the sampled layer rather than direct evidence of leaching losses. In contrast, Zn showed evidence of surface accumulation, reflecting its higher cumulative input and intermediate affinity for soil constituents. The contrasting behavior between Zn and Cu may be partially associated with differences in their interaction with soil constituents. Copper generally shows stronger affinity for organic matter and mineral surfaces, which may contribute to lower detectable changes in pseudo-total concentrations compared with Zn.

Dolomitic limestone influences nutrient dynamics by increasing soil pH, which can enhance vertical distribution of elements such as Cd, Ni, Cr, and Pb. However, in our study, the maximum soil pH recorded was 5.38, a relatively low level where PTEs remain highly available in the soil solution. The absence of significant limestone effects in this study suggests that the observed pH changes were not sufficient to produce detectable alterations in pseudo-total PTE concentrations during the evaluated period. In contrast, pH values closer to neutral (pH ~ 7) are generally required for effective retention via Fe/Mn oxyhydroxide interactions (Madrid and Marrugo, 2021).

The cumulative load approach provides additional insight into the contrasting behavior among elements. Although all PTE concentrations remained below regulatory thresholds, Zn inputs from poultry litter represented the largest proportional contribution relative to the initial soil stock. This result helps explain why Zn was the only element showing a consistent dose-dependent response after repeated applications. Conversely, the relatively small contribution of As, Cd, Cr, and Ni inputs compared with their initial soil pools may partly explain the absence of detectable accumulation during the evaluated period.

Poultry litter contributes to increased organic matter and phosphorus, facilitating the formation of soluble metal–organic complexes, i.e., PTE retention and displacement due to competitive adsorption, a process supported by higher soil pH values (Bolan et al. 2004; Antoniadis et al. 2019; Jaja et al. 2023). Underpinning this idea, soils fertilized with poultry litter showed an average increase in organic matter content and phosphorus in surface layers up to 20 cm (Mitchell and Tu 2006). Nevertheless, the release and bioavailability of PTEs may occur through the mineralization of organic matter (De Souza et al. 2019), largely affecting less fixed elements linked with organic matter. Consistent with our findings, previous studies have also reported no significant accumulations of heavy metals with the application of poultry (Mitchell and Tu 2006; Xiao et al. 2022). Consequently, PTEs introduced by poultry litter to the soil could have been retained by organic matter and displaced to deeper layers by the increase in phosphorus, without a PTE-reducing effect due to the low pH values of the soil during the trial.

Natural factors influencing Cd levels in soils include pH, clay content, topography, rock weathering, soil erosion, and leaching (Ballabio et al. 2024). However, anthropogenic phosphorus inputs due to abundant fertilizer usage cause competition for adsorption sites in the soil, displacing and promoting cadmium bioavailability (Ballabio et al. 2024). Moreover, Cd in soils with acidic conditions tends to be available in the soil solution (Dziubanek et al. 2017), showing a high soil–plant transfer potential for having more soluble soil fractions (Antoniadis et al. 2019). As a result, Cd vertical distribution in soils may be limited and absorbed by crops (Ballabio et al. 2024). In the present study, the limited pH increase after amendment application may have contributed to maintaining conditions favorable to Cd mobility. However, Cd redistribution to deeper soil layers or plant uptake was not directly evaluated and should be investigated in future studies. Nickel (Ni) was found to be soluble in poultry litter (Jackson et al. 2003). The amendment addition to organic-rich soils enhances the reduction or transformation and reduces the availability of certain metals such as Cr, Ni, and Se (Bolan et al. 2004). Hence, low pH in the soil may not have influenced Cr or Ni fixation and supported leaching to lower soil depths.

We have found no significant increases in extractable Cu in soils amended with pig manure or poultry litter for up to 10 years (Brock et al. 2006; Jaja et al. 2022; Xiao et al. 2022). However, small amounts of soluble Cu were added to the soil because of its organic complexity (Bolan et al. 2004). Other parameters, such as high pH, soil organic carbon, and clay content, with humid and wet climatic conditions, increase the accumulation of Cu in soils (Ballabio et al. 2018). According to Parente et al. (2019), poultry litter application sharply increased Cu concentration in the soil’s upper layer (0–10 cm). In this study, organic matter additions from poultry litter may have contributed to Cu retention through complexation processes, potentially explaining the limited changes observed in pseudo-total Cu concentrations. However, chemical fractionation studies are necessary to confirm the mechanisms controlling Cu dynamics under repeated poultry litter applications.

Zinc showed clear surface accumulation (0–10 cm) under high poultry litter doses (8–12 t ha⁻^1^), reaching 64.37 mg kg⁻^1^. This reflects Zn’s intermediate organic affinity—strong enough to retain it in surface layers via litter-derived organic matter, but weak enough to prevent deep leaching. Several studies have reported high concentrations of this element in soil when receiving 15 to 40 years of poultry litter applications (Ashjaei et al. 2011; Ashworth and Moore 2023; Foust et al. 2018; Jackson et al. 2003; Jaja et al. 2023; Mitchell and Tu 2006; Xiao et al. 2022). Although concentrations remained below toxicity thresholds, the dose-dependent pattern suggests progressive buildup with continued applications. Also, Zn is more available in soil solutions because the complex formation with organic matter is weaker than other cations, although there is no evidence that Zn accumulation in the plow layer has reached toxicity thresholds, even after 40 years of manure applications (Brock et al. 2006). Thus, this agricultural input allowed the increase of phosphorus and organic matter in the first soil depths, and the constant and large addition of Zn may lead to an increase in the concentrations on the topsoil surface.

It is important to acknowledge that soil PTE concentrations were evaluated until 2018, representing eight consecutive years of poultry litter application. Therefore, potential changes occurring after this period cannot be excluded, particularly if organic amendments continue to be applied over longer timescales. Continued monitoring, including deeper soil layers, chemical fractionation approaches, and plant uptake assessments, is necessary to better understand the long-term environmental implications of cumulative PTE inputs. Finally, while Brazilian regulatory thresholds were not exceeded, the proximity of As and Cd concentrations to more restrictive international guidelines raises questions about the adequacy of current reference values for long-term soil quality protection, particularly under cumulative organic amendments. However, more stringent regulations, such as those in the USA and the European Union, which set lower tolerable limits for As, Cd (Ballabio et al. 2024), and Cr, indicate that contamination may occur for these elements. PTEs can be released into the soil from different sources, such as i) weathering from parent rocks and ii) the application of pesticides and fertilizers. The presence of PTEs can be mitigated through their natural detection in the soil and exhaustive monitoring of the amounts added by the consecutive use of poultry litter.

## Conclusion

This eight-year field study demonstrates that under agronomically realistic poultry litter application rates, medium-term changes in pseudo-total concentrations of most potentially toxic elements are difficult to detect in an acidic subtropical soil. No statistically significant accumulation of As, Cd, Cr, Cu, or Ni was detected within the evaluated soil layers and experimental period, despite continuous inputs over eight years. These findings may reflect the relatively low cumulative inputs of these elements compared with initial soil stocks and potential retention mechanisms associated with soil properties. In contrast, Zn consistently accumulated in the surface layer (0–10 cm), exhibiting a clear dose-dependent response to poultry litter application. This pattern identifies Zn as a sensitive indicator of accumulation and a priority element for environmental monitoring in systems receiving repeated organic amendments. Dolomitic limestone had no detectable influence on PTE dynamics, likely because the pH increase achieved was insufficient to substantially alter metal retention processes. Although all PTE concentrations remained below Brazilian regulatory thresholds, the proximity of As and Cd levels to more restrictive international guideline values raises questions about the adequacy of current reference limits for long-term soil quality protection. Overall, the study highlights the importance of baseline soil characterization, realistic mass balance interpretation, and cautious assessment of medium-term field data when evaluating environmental risks associated with poultry litter application. Continued monitoring, deeper soil sampling, and integration with plant uptake data are recommended to support more comprehensive risk assessments in tropical agroecosystems.

## Supplementary Information

Below is the link to the electronic supplementary material.

ESM 1 (DOCX 15.8 KB)

ESM 2 (DOCX 26.0 KB)

## Data Availability

The datasets generated during the current study are available from the corresponding authors upon reasonable request. R code is available at GitHub < https://github.com/masemuta > to reproduce statistical analyses.
