# Supplementary material for: Medium-term impacts of poultry litter application on potentially toxic elements in soil under subtropical conditions in southern Brazil
Source: Environ Sci Pollut Res Int. 2026 Jun 30;33(20):10266–76. doi: 10.1007/s11356-026-37995-3 (PMC13350140; doi:10.1007/s11356-026-37995-3)
Supplement: Supplementary file 1 — (DOCX 15.8 KB) [file 11356_2026_37995_MOESM1_ESM.docx]

**Supplementary Table S1.** Reference pseudo-total potentially toxic element (PTE) concentrations in the absolute control treatment (without poultry litter and dolomitic limestone application) during the 2017 and 2018 sampling years. Values are provided as background reference concentrations for contextual interpretation.

| Elements  (mg kg^-1^) | As | Cd | Cr | Cu | Ni | Zn | pH-CaCl_2_  CT MD* | |
| --- | --- | --- | --- | --- | --- | --- | --- | --- |
| Year | 2017 | | | | | | | |
| 0-10 cm | 9.6 | 0.8 | 53.3 | 56.4 | 9.7 | 44.7 | 4.3 | 5.0 |
| 10-20 cm | 14.3 | 0.7 | 43.1 | 77.5 | 16.5 | 42.2 | 4.5 | 4.6 |
| Year | 2018 | | | | | | | |
| 0-10 cm | 10.5 | 0.9 | 41.2 | 68.3 | 9.6 | 43.3 | 4.5 | 5.4 |
| 10-20 cm | 12.4 | 0.8 | 42.9 | 67.0 | 9.7 | 48.8 | 4.5 | 4.5 |

MD*: pH quantified in soil with the addition of 8 t ha^-1^ of dolomitic limestone and 12 t ha^-1^ of poultry litter.
