# Supplementary material for: Medium-term impacts of poultry litter application on potentially toxic elements in soil under subtropical conditions in southern Brazil
Source: Environ Sci Pollut Res Int. 2026 Jun 30;33(20):10266–76. doi: 10.1007/s11356-026-37995-3 (PMC13350140; doi:10.1007/s11356-026-37995-3)
Supplement: Supplementary file 2 — (DOCX 26.0 KB) [file 11356_2026_37995_MOESM2_ESM.docx]

**Supplementary Table S2.** Complete dataset of pseudo-total potentially toxic element (PTE) concentrations (mg kg⁻¹; mean ± standard error) according to sampling year (2017 and 2018), amendment source (dolomitic limestone and poultry litter), application rate, and soil depth. Different letters indicate significant differences according to Tukey’s HSD test (p < 0.05).

| **Source** | **Dose (t ha⁻¹)**  **2017 year** | **Depth (cm)** | | **PTE (mg kg⁻¹) – 2017** | | | | | | | | | | | |
| --- | --- | --- | --- | --- | --- | --- | --- | --- | --- | --- | --- | --- | --- | --- | --- |
|  |  |  |  | **As** | | **Cd** | | **Cr** | | **Cu** | | **Ni** | | **Zn** | |
| **Dolomitic limestone** | **0** | **0-10** | | 14.03±0.51ª | | 0.79±0.06ª | | 40.40±1.76ª | | 75.26±1.68ª | | 16.22±0.94ª | | 61.00±3.86ª | |
|  |  | **10-20** | | 13.76±0.41ª | | 0.79±0.07ª | | 42.25±2.15ª | | 79.09±2.74ª | | 15.28±0.71ª | | 43.44±2.50ª | |
|  |  | **Mean** | | ***13.89±0.34ª*** | | ***0.79±0.06ª*** | | ***41.37±1.45ª*** | | ***77.17±1.69ª*** | | ***15.75±0.58ª*** | | ***52.22±2.57ª*** | |
|  | **2** | **0-10** | | 15.25±0.39ª | | 0.94±0.03ª | | 39.27±1.37ª | | 82.26±2.09ª | | 14.20±1.02ª | | 62.58±4.20ª | |
|  |  | **10-20** | | 13.96±0.70ª | | 0.93±0.06ª | | 40.90±1.52ª | | 80.19±4.07ª | | 15.60±1.43ª | | 42.31±3.39ª | |
|  |  | **Mean** | | ***14.61±0.38ª*** | | ***0.93±0.06ª*** | | ***40.08±1.52ª*** | | ***81.22±2.72ª*** | | ***14.9±0.970ª*** | | ***52.45±3.24ª*** | |
|  | **4** | **0-10** | | 15.08±1.02ª | | 0.90±0.09ª | | 41.61±2.76ª | | 74.29±2.89ª | | 16.43±2.10ª | | 60.66±5.39ª | |
|  |  | **10-20** | | 15.65±0.40ª | | 1.01±0.08ª | | 45.94±1.31ª | | 76.08±2.52ª | | 19.27±2.52ª | | 50.04±2.74ª | |
|  |  | **Mean** | | ***15.36±0.44ª*** | | ***0.96±0.07ª*** | | ***43.78±1.86ª*** | | ***77.15±2.60ª*** | | ***17.85±0.78ª*** | | ***55.35±3.65ª*** | |
|  | **8** | **0-10** | | 14.93±0.80ª | | 1.09±0.08ª | | 44.24±2.09ª | | 83.56±3.81ª | | 15.25±0.60ª | | 61.73±4.16ª | |
|  |  | **10-20** | | 14.36±0.23ª | | 1.00±0.05ª | | 40.95±0.58ª | | 80.02±1.75ª | | 14.80±0.35ª | | 44.64±2.56ª | |
|  |  | **Mean** | | ***14.64±0.40ª*** | | ***1.05±0.05ª*** | | ***42.59±1.01ª*** | | ***81.79±2.17ª*** | | ***15.02±0.32ª*** | | ***53.18±2.72ª*** | |
| **Poultry litter** | **0** | **0-10** | | 13.83±0.99ª | | 0.89±0.10ª | | 39.30±2.61ª | | 73.03±2.62ª | | 14.26±1.03ª | | 47.10±3.77ª | |
|  |  | **10-20** | | 14.69±0.61ª | | 0.92±0.07ª | | 40.77±1.08ª | | 77.17±4.30ª | | 15.16±0.85ª | | 42.46±3.03ª | |
|  |  | **Mean** | | ***14.54±0.48ª*** | | ***0.91±0.08ª*** | | ***40.04±1.34ª*** | | ***77.07±2.99ª*** | | ***14.70±0.60ª*** | | ***44.78±2.62ᵇ*** | |
|  | **4** | **0-10** | | 15.10±0.43ª | | 0.85±0.05ª | | 40.61±1.85ª | | 79.37±2.99ª | | 14.49±1.17ª | | 56.39±3.11ª | |
|  |  | **10-20** | | 14.41±0.44ª | | 0.90±0.07ª | | 43.46±1.51ª | | 78.97±2.86ª | | 17.74±2.43ª | | 45.76±2.52ª | |
|  |  | **Mean** | | ***14.75±0.24ª*** | | ***0.87±0.05ª*** | | ***42.03±1.54ª*** | | ***79.17±2.53ª*** | | ***16.12±0.78ª*** | | ***51.07±2.49ªᵇ*** | |
|  | **8** | **0-10** | | 15.49±0.79ª | | 1.00±0.07ª | | 44.21±2.14ª | | 83.84±3.57ª | | 16.78±0.91ª | | 70.51±3.65ᵇ | |
|  |  | **10-20** | | 14.19±0.56ª | | 0.98±0.07ª | | 41.54±1.14ª | | 79.47±1.72ª | | 15.13±0.64ª | | 48.09±3.63ª | |
|  |  | **Mean** | | ***14.84±0.54ª*** | | ***0.99±0.06ª*** | | ***42.87±1.29ª*** | | ***81.66±2.07ª*** | | ***15.95±0.54ª*** | | ***59.30±3.30ª*** | |
|  | **12** | **0-10** | | 14.86±0.50ª | | 0.98±0.06ª | | 41.50±1.48ª | | 79.13±1.68ª | | 16.58±1.82ª | | 71.98±3.67ᵇ | |
|  |  | **10-20** | | 14.43±0.38ª | | 0.92±0.08ª | | 44.26±2.07ª | | 79.76±2.07ª | | 16.93±1.55ª | | 44.11±2.06ª | |
|  |  | **Mean** | | ***14.65±0.35ª*** | | ***0.95±0.06ª*** | | ***42.88±1.82ª*** | | ***79.44±1.64ª*** | | ***16.75±0.83ª*** | | ***58.04±2.29ª*** | |
| **Source** | **Dose (t ha⁻¹)**  **2018 year** | | **Depth (cm)** | | **PTE (mg kg⁻¹) – 2018** | | | | | | | | | | |
|  |  |  |  |  | **As** | | **Cd** | | **Cr** | | **Cu** | | **Ni** | | **Zn** |
| **Dolomitic limestone** | **0** | | **0-10** | | 11.68±0.51ª | | 0.73±0.06ª | | 32.25±3.19ª | | 67.92±3.76ª | | 9.65±1.25ª | | 51.40±3.59ª |
|  |  |  | **10-20** | | 10.87±0.57ª | | 0.62±0.07ª | | 30.75±3.35ª | | 70.23±3.51ª | | 9.89±1.40ª | | 43.96±3.26ª |
|  |  | | **Mean** | | ***11.27±0.59ª*** | | ***0.68±0.06ª*** | | ***31.50±3.23ª*** | | ***69.07±2.94ª*** | | ***9.77±1.23ª*** | | ***47.68±2.95ª*** |
|  | **2** | | **0-10** | | 12.32±0.52ª | | 0.63±0,06ª | | 34.64±2.94ª | | 76.17±3.43ª | | 13.95±1.19ª | | 48.30±3.65ª |
|  |  |  | **10-20** | | 12.52±0.45ª | | 0.73±0.08ª | | 38.07±3.48ª | | 77.84±2.36ª | | 14.42±1.18ª | | 46.61±2.64ª |
|  |  | | **Mean** | | ***12.41±0.44ª*** | | ***0.68±0.06ª*** | | ***36.35±3.07ª*** | | ***77.00±1.84ª*** | | ***14.18±1.06ª*** | | ***47.46±2.69ª*** |
|  | **4** | | **0-10** | | 11.64±0.42ª | | 0.64±0.08ª | | 31.14±3.24ª | | 66.31±2.07ª | | 8.68±0.84ª | | 43.21±2.66ª |
|  |  |  | **10-20** | | 11.51±0.62ª | | 0.58±0.08ª | | 31.38±3.36ª | | 63.87±2.61ª | | 8.02±1.29ª | | 36.38±2.01ª |
|  |  | | **Mean** | | ***11.57±0.66ª*** | | ***0.61±0.07ª*** | | ***31.25±3.19ª*** | | ***65.08±2.20ª*** | | ***8.35±1.00ª*** | | ***39.79±1.78ª*** |
|  | **8** | | **0-10** | | 12.50±0.35ª | | 0.63±0.06ª | | 32.11±1.77ª | | 67.10±2.39ª | | 10.37±0.81ª | | 46.45±4.21ª |
|  |  |  | **10-20** | | 12.53±0.57ª | | 0.59±0.04ª | | 33.13±2.21ª | | 67.50±2.24ª | | 10.64±0.84ª | | 39.16±2.49ª |
|  |  | | **Mean** | | ***12.52±0.51ª*** | | ***0.61±0.04ª*** | | ***32.62±1.63ª*** | | ***67.30±2.14ª*** | | ***10.50±0.69ª*** | | ***42.80±2.88ª*** |
| **Poultry litter** | **0** | | **0-10** | | 11.67±0.38ª | | 0.66±0.07ª | | 32.04±3.14ª | | 65.90±2.69ª | | 10.00±0.97ª | | 37.12±1.58ª |
|  |  |  | **10-20** | | 12.53±0.62ª | | 0.68±0.07ª | | 35.57±2.69ª | | 68.34±3.81ª | | 10.70±1.46ª | | 41.69±2.81ª |
|  |  | | **Mean** | | ***12.10±0.55ª*** | | ***0.67±0.07ª*** | | ***33.80±2.91ª*** | | ***67.12±2.90ª*** | | ***10.36±1.16ª*** | | ***39.40±2.01ª*** |
|  | **4** | | **0-10** | | 12.61±0.55ª | | 0.67±0.08ª | | 33.14±3.46ª | | 75.00±2.84ª | | 12.52±1.06ª | | 47.87±3.29ª |
|  |  |  | **10-20** | | 11.30±0.46ª | | 0.65±0.07ª | | 32.71±2.84ª | | 74.65±1.85ª | | 12.08±1.02ª | | 42.71±2.54ª |
|  |  | | **Mean** | | ***11.95±0.53ª*** | | ***0.66±0.06ª*** | | ***32.92±2.74ª*** | | ***74.82±1.78ª*** | | ***12.30±0.85ª*** | | ***45.30±2.72ª*** |
|  | **8** | | **0-10** | | 12.00±0.45ª | | 0.63±0.06ª | | 30.94±3.02ª | | 65.19±2.63ª | | 9.22±1.35ª | | 47.59±3.21ª |
|  |  |  | **10-20** | | 11.59±0.66ª | | 0.55±0.05ª | | 31.41±2.63ª | | 66.52±2.78ª | | 9.57±1.37ª | | 39.50±3.13ª |
|  |  | | **Mean** | | ***11.80±0.47ª*** | | ***0.59±0.05ª*** | | ***31.17±2.93ª*** | | ***65.86±2.54ª*** | | ***9.39±1.24ª*** | | ***43.55±2.70ª*** |
|  | **12** | | **0-10** | | 14.88±0.43ª | | 0.68±0.06ª | | 34.00±3.16ª | | 71.39±2.50ª | | 10.90±1.06ª | | 56.76±4.10ª |
|  |  |  | **10-20** | | 11.99±0.53ª | | 0.65±0.07ª | | 33.63±3.62ª | | 69.92±1.06ª | | 10.61±1.38ª | | 42.19±2.74ª |
|  |  | | **Mean** | | ***11.93±0.54ª*** | | ***0.66±0.06ª*** | | ***33.81±2.98ª*** | | ***70.66±2.27ª*** | | ***10.75±1.20ª*** | | ***49.48±2.84ª*** |
